# Turnaround Time: An Efficacy Measure for Medical Laboratories

**DOI:** 10.7759/cureus.28824

**Published:** 2022-09-06

**Authors:** Pratibha P Dawande, Rashmi S Wankhade, Faizan I Akhtar, Obaid Noman

**Affiliations:** 1 Pathology, Jawaharlal Nehru Medical College, Datta Meghe Institute of Medical Sciences, Wardha, IND

**Keywords:** tat, benchmark, post-analytical phase, analytical, pre-analytical, turn around time

## Abstract

Turnaround time (TAT), which doctors frequently use as the benchmark for laboratory performance, is a typical way to communicate timeliness. It also acts as a quality indicator to evaluate the effectiveness and efficiency of the testing process and the satisfaction of clinicians and patients. TAT is the time from receipt of the sample in the laboratory to final delivery or dispatch of the report of said test. The TAT procedure can be broadly divided into three stages pre-analytical, analytical and post-analytical. There is variability in TAT according to different conditions like the volume of sample size, staff expertise, availability of adequate resources, distances of the hospital from the lab, and various sub-departments. To remove obstacles to optimizing TAT, we must take a practical approach. A workload reduction plan, proper stock management, specialized work assignments, and skilled staff retention are crucial strategies to reduce the setting's delayed TAT.

## Introduction and background

Multi-specialty hospitals are the backbone of the health care system globally, corporate or educational institutional hospitals. While catering needs of different domains of patients like medical, surgical, orthopedics, pediatrics, obstetrics, and gynecology or a super-specialty branch, an in-house clinical laboratory makes the backbone of all these health care services. A Central Clinical Laboratory is mandatory for educational institutes and hospitals as per notification of the National Medical Commission. Likewise, it has been found most feasible for non-institutional health care services (like corporate multi-specialty hospitals) to have an in-house laboratory service.

While internal and external quality control measures check the quality of the said test (IQAP and EQAP), “turnaround time” (TAT) is one of the best assessors of efficient laboratory performance. Laboratory experts may overlook the time-bound outcome of any laboratory test to the analytical accuracy of the test. But clinical experts need proper time-bound reports for diagnostic and therapeutic decisions. In a surgical pathology lab, TAT can be affected by various factors, including tissue type, number of slides, decalcification, immunohistochemistry, and secondary consultations with co-pathologists [1].

Accordingly, TAT will be defined as the time from receipt of the sample in the laboratory to final delivery or dispatch of the report of said test. TAT is “A parameter of a clinical laboratory's efficiency, defined as the time between ordering a test or submitting a specimen to the lab to reporting results” [2]. Though the term was initiated in the shipping and airline industry, it is routinely used in medical laboratories, which implies the time taken to complete the test [3].

Quality is the capacity of a good or service to meet the requirements and expectations of the user. The concept of quality in laboratories has typically been limited to technical or analytical quality, concentrating on imprecision and inaccuracy objectives. However, clinicians are more concerned with service quality, which includes availability, cost, relevance, and timeliness in addition to total test error (imprecision and inaccuracy). Clinicians desire a quick, dependable, and cost-effective solution. Timeliness is likely the most significant of these qualities to the doctor, who may be willing to forego analytical quality in exchange for a quicker TAT. Much of the current growth of point-of-care testing (POCT) is motivated by this inclination [4].

The pathology laboratory samples are processed and analyzed after appropriate techniques depending on the type of sample or specimen. Most frequently, a clinician's doubt regarding the presence or extent of malignancy leads to the choice to perform a biopsy on a patient. As a result, doctors and their patients expect surgical pathology reports to be accurate and delivered quickly. Economic concerns that aim to shorten hospital stays and settle costs quickly after release adds to the need for a quick TAT in surgical pathology. So, a surgical pathology laboratory's TAT is a crucial indicator of its quality. TATs are essential in clinical pathology as well. For instance, rapid TATs for tests like troponin and creatinine kinase-MB greatly aid in the early diagnosis and treatment of patients with acute myocardial infarction. Additionally, rapid TATs for laboratory tests generally shorten the time of stay for patients in emergency rooms [5].

## Review

Different phases in the TAT procedure 

The TAT procedure can be broadly divided into three phases - pre-analytical, analytical, and post-analytical. 

**Table 1:** Broad categorization of TAT

Pre-analytical	Analytical	Post- analytical
Test Order	Segregation	Interpretation
Collection	Analysis	Action
Transport	Reporting	

Pre-analytical Phase

It starts when the consultant orders a test to sample received in the laboratory. Let us consider the process of reception of samples in most laboratories. After the consultant’s advice, outpatient department patients are usually directed to a phlebotomy center to give blood samples. Phlebotomy centers are mostly (and should ideally be) situated in Central Clinical Laboratory. The clinical or para-clinical staff collect blood samples for indoor patients in various wards, e.g., junior/senior residents, or nursing staff. To avoid delay in this pre-analytical phase of sample collection, hospitals need to have an in-house clinical laboratory. A central clinical laboratory away from the hospital will pose obstacles for the sample to reach the laboratory in time and may affect results. It may also be challenging for patients to get to phlebotomy centers by themselves to give blood samples and collect reports, primarily for pediatric, geriatric, and pregnant patients.

The efficiency of phlebotomists also matters in collecting and sending samples to the laboratory. Pneumatic tubes help to reduce this time remarkably. Being too costly, provision may not be feasible and possible for all hospitals. The samples of outpatient department patients are collected in batches and processed. The time allotted for each batch collection depends on the number of patients, i.e., one or two hours. This time delays TAT in routine samples. Stat or emergency samples are immediately taken for processing.

After the sample reaches the laboratory from the ward or phlebotomy center, details of patients are entered into a system/documented in registers. These details include patient demographics, e.g., age sex, address, registration number, clinical details, and bill payment receipt. Employing a Laboratory Information System (LIS) can be time-saving for this process. As we understand, tests can be advised on a routine basis (especially in the outpatient department) or can be STAT for indoor admission. It can be on an emergency basis, especially in intensive care wards. TAT varies in all such situations. Central Clinical laboratories usually have sections covering “Pathology, Microbiology, Biochemistry” work. Few laboratories also include sections in Surgical Pathology, Cytology, and Special hematology. Sample distribution is a critical step in ensuring proper workflow. An expert and efficient hand is needed for such work.

There are quite a few variations in the measurement of TAT which can complicate comparison at different laboratories. The process is made more difficult by several consecutive cycles, each of which has a minimum or maximum time constraint and delays or problems. Thus, the Gaussian distribution in each of the individual steps is not as expected, making the use of standard deviation as inappropriate as TAT descriptors [4]. An alternative strategy is to investigate the TAT using a Kaplan-Meier plot and failure time analysis, which can be helpful [5]. The ideal metrics are the median and tall size, and it is thought that a non-gaussian distribution and a positive skew are more acceptable. Valenstein and Emancipator studied these measures of TAT in detail in their study [6].

Analytical Phase

After the segregation of samples to respective workstations, actual analytical work begins. It includes separation, analysis, and reporting. While a laboratory expert is more conscious of precise and quality research, a clinician is most concerned about the time taken to report samples, especially when it is a therapeutic test [7]. Nowadays, in view of the increasing demand for point of care (POC), TAT has evolved into one of the most recognizable indicators of laboratory service, and many physicians use them to assess the level of a laboratory's efficiency [8,9].

Different tests have variable TAT requirements, e.g., the prothrombin time is to be performed immediately and conveyed to the clinician, while other test reports can be delayed for a while. Outpatient department patients may collect reports the next day at their convenience. Here delay of TAT is because of the lag post-analytical phase. Different laboratories have a distinct set of equipment resulting in variations of TAT. Such equipment, to be cost-effective, need a significant focus on maintenance. Any failure or breakdown of equipment can lead to a delay of TAT for multiple tests.

Post-analytical Phase

This includes approval of reports by laboratory consultants, entering reports in the system, or documenting in the register where the LIS is unavailable. TAT is improved when approved reports are entered into the LIS, which can be seen on the system by the other side, i.e.., clinicians on the LIS. Hard copies of such statements should be printed at the clinician’s end only.

Outpatient department/routine reports can also have similar access. At some hospitals, outpatient department patients collect their reports in hard copies and show them to their respective clinicians. In such situations, the time taken by patients to compile reports at their convenience will lead to variable TAT.

Effects of delayed TAT

The laboratory must deliver accurate, dependable, and prompt test results to guarantee high-quality diagnostic services. TAT, which doctors frequently use as the benchmark for laboratory performance, is a typical way to communicate timeliness. It also acts as a quality indicator to evaluate the effectiveness and efficiency of the testing process and the satisfaction of clinicians and patients [10-13]. Timely reporting is essential for making appropriate health care decisions that affect patient outcomes. Therefore, a quick TAT in the laboratory is crucial to handle patient care promptly. Consequently, this period aids in identifying the reason for the delay [14].

When there is a delay, quality improvements must comprehend the primary causes of high TAT using evidence-based methodologies. As a result, regulatory and accrediting organizations advise clinical laboratories to focus on TAT in their strategy for continuous improvement. Delays in reporting, and laboratory results would delay the diagnosis and treatment of patients. Research revealed a 61% longer stay in the emergency room and a 43% treatment delay [15,16].

A sluggish TAT can also result in a rise in requests, which duplicates the test [17]. This adds to the effort in the laboratory and could increase the cost of health care [18].

Causes of delayed TAT

Other studies have shown equipment breakage as the most frequent cause of laboratory result delays, followed by issues with reagent stock out, machine maintenance and technical staff oversight, and having more test options. Other causes of a delayed TAT include the failure to modify work schedules to coordinate available manpower and a lack of manpower [4]. Additionally, there were activity overlaps caused by supportive supervision, mentorship, and intensive training provided to peripheral laboratories which kept laboratory staff busier and could have delayed the TAT of patient data [19,20].

A study done in Malawi also revealed that the specimen's origin, kind, and testing facility were primarily related to the viral load test's prolonged TAT [21].

The TAT for laboratory results in Animal Population Health Institute (APHIS) was delayed. The most impacted tests that require improvement are those for HIV viral load, early infant diagnosis, and TB gene expert findings. To reduce the setting's delayed TAT, workload reduction plans, proper stock management, particular work assignments, and skilled staff retention are crucial strategies [22].

Benefits of TAT control

· Optimized TAT benefits your daily revenue and increases your monthly revenue cycle on an operational level.

· You get more confidence from clients, referrals, and business partners when you deliver reports quickly and reliably.

· Improved service delivery times also result in a rise in the number of samples processed daily. With a reduced delivery period, monthly throughput can be doubled.

· Physicians can respond to reports more quickly, thanks to shortened reporting times. The patient service time is improved. As a result, this timeline optimization enhances doctors' and patients' interactions with the diagnostic facility. These tangible and nontangible benefits give your laboratory center the chance to grow [22].

Improvement of TAT 

To successfully meet the goals of improving TAT, many measures can be undertaken at various steps affecting the final TAT, from the pre-analytical to the final post-analytical phases. The use of technological advancement can significantly help in improving the TAT. We summarize the various measures that can be taken to improve the TAT in Table 2.

**Table 2:** TAT improvement measures LIS: Laboratory Information System

Step	Measures to improve TAT
Test order	Use the LIS system Standardize test nomenclature
Specimen collection and transport	Ensure a proper system of collection of patient details for traceability. Proper labeling of sample/specimen. Using trained personnel for phlebotomy/sample collection. Ensuring accuracy of data entered by the clinical staff. Keeping a record of transport conditions and accountability of staff assigned.
Accessioning	Use of bar codes Use of pneumatic tubes or conveyer belt system. The skilled, trained staff at accessioning department.
Testing	Use of automation systems wherever possible. Regular checkups and maintenance of instruments. Automatic verification of results within average reference range values. Daily running of quality control and keeping records of the same
Reporting	Interfacing of machine software with LIS system. Training/CME conducted by and for pathologists. Use LIS to trace relevant/other tests of the same patient for correlation to improve reporting accuracy. Release of reports directly to the clinician through the LIS. Automation of process wherever applicable to reduce manual input errors.
General	Monitoring of TAT daily and analysis of the same promptly. Tracking errors and implementing solutions to eliminate them.

The physicians rely on laboratory services to implement and assess treatment modalities. Therefore, it is within our purview to guarantee promptness. The findings of our investigation make it clear that there is much room for improving TAT in our setting. Over the past few years, there has been a significant shift in how clinical biochemists perceive laboratory effectiveness. We know that the pre-analytical and analytical phases are crucial for laboratories, particularly in TAT [23]. The researchers have provided numerous descriptions of TAT. The nine steps of the TAT are ordering, collecting, identification, transport, preparation, analysis, reporting, interpretation, and action, according to the "total testing cycle” [24].

The adjective therapeutic TAT refers to the period between the request for a test and the decision to proceed with treatment. Depending on the various stages of sample processing, TAT can be categorized as pre-analytical, analytical, and post-analytical [4].

The average TAT for Emergency Room and ICU departments as well as outpatient department and indoor departments was 1 hour and 3 minutes and 1 hour and 21 minutes, respectively, according to a detailed study for specific departments. Only 2.03% of the state samples in the study by Bilwani et al. had TAT longer than the allowed range [14].

It was discovered that the analytical and post-analytical phases of the test experienced the most extraordinary TAT delay which was greater than 60 minutes, during the morning shift. The analysis or machine breakdown was determined to be the most frequent cause of this delay, followed by machine maintenance and technical staff negligence issues. This contrasted the grounds of the delay in the analytical phase reported by previous investigations. These delays have been attributed to the most significant single factor: a shortage of highly qualified staff. Other factors that contribute to pre-analytical delays, such as receipt to verification times, include technical issues with instruments, specimen issues with results that need to be verified, laboratory accidents, and administrative matters involving data entry, among others [25].

Pre-analytic TAT is said to increase during the day according to a College of American Pathologists Q-Probes Study; however, this may be due to transit and collecting stage delays [20].

To obtain the fastest TAT possible, each phase's pre-analytical and post-analytical steps can be accelerated in various ways. A ground-breaking invention that has transformed sample transport is the pneumatic system. Numerous studies have demonstrated the effectiveness of this approach in minimizing unintended delays caused by human couriers [26].

Optimum phlebotomy techniques, bar coding of samples, and computer-generated requisition slips are other ways to reduce pre-analytical delays. These procedures will lessen the uncertainties brought on by illegible mistakes and poor selection collecting methods. The uncertainties brought on by centrifugation can be minimized by using gel vacutainers. Complete laboratory automation, the use of equipment with higher throughout, the use of plasma or whole blood samples, primary tube sampling, ensuring minimal downtime and having an adequate backup, implementing effective quality control procedures, using automatic dilutions when results are not linear, quickly validating reports, etc. can all speed up the analytical phase. Additionally, it is crucial to ensure that the technicians have a good division of labor so that sample processing and reporting go smoothly. The team should receive training on expeditiously processing urgent samples while handling them with the utmost care [4,27,28].

Adopting laboratory information services can significantly improve the post-analytical process. By doing this, transcriptional errors and delays in report delivery to the appropriate wards will be eliminated. In a circumstance of understaffed laboratories, adding more staff to work can speed up the delivery of the report [28].

Another tactic is to advise the patients as soon as possible about fundamental values and pre-analytical mistakes so that repeat samples can be processed quickly. Creating an open and efficient communication system between clinicians and laboratory technicians is crucial [20].

## Conclusions

TAT has been a cornerstone for measuring laboratory efficiency in recent years. Despite the advances in the technology, transport, and training of staff, TAT remains a cause of patient and clinician dissatisfaction. There is variability in TAT according to different conditions like the volume of sample size, staff expertise, availability of adequate resources, distances of the hospital from the lab, and various sub-departments. Also, it’s difficult to get exact and precise measurements making it very challenging to maintain optimum TAT. To remove obstacles to optimizing TAT, we must take a practical approach. To reduce delayed TAT, a workload organization plan, proper stock management, specialized work assignments, and skilled staff retention are crucial strategies.
